# Natural language processing for detecting adverse drug events: A systematic review protocol

**DOI:** 10.3310/nihropenres.13504.3

**Published:** 2024-12-10

**Authors:** Imane Guellil, Jinge Wu, Aryo Pradipta Gema, Farah Francis, Yousra Berrachedi, Nidhaleddine Chenni, Richard Tobin, Clare Llewellyn, Stella Arakelyan, Honghan Wu, Bruce Guthrie, Beatrice Alex

**Affiliations:** 1 The University of Edinburgh, Edinburgh, Scotland, UK; 2 University College London, London, England, UK; 3 Ecole nationale Superieure d'Informatique,ESI, Alger, Algiers, Algeria

**Keywords:** Systematic review protocol, systematic review, Adverse Drug Events, side effect, Natural Language Processing

## Abstract

**Background:**

Detecting Adverse Drug Events (ADEs) is an emerging research area, attracting great interest in the research community. Better anticipatory management of predisposing factors has considerable potential to improve outcomes. Automatic extraction of ADEs using Natural Language Processing (NLP) has a great potential to significantly facilitate efficient and effective distillation of such knowledge, to better understand and predict risk of adverse events.

**Methods:**

This systematic review follows the six-stage including the literature from 6 databases (Embase, Medline, Web Of Science Core Collection, ACM Guide to Computing Literature, IEEE Digital Library and Scopus). Following the title, abstract and full-text screenings, characteristics and main findings of the included studies and resources will be tabulated and summarized. The risk of bias and reporting quality was assessed using the PROBAST tool.

**Results:**

We developed our search strategy and collected all relevant publications. As of December 2024, we have completed all the stages of the systematic review. We identified 178 studies for inclusion through the academic literature search (where data was extracted from all of the papers). Right now, we are writing up the systematic review paper where we are synthesising the different findings. Further refinement of the eligibility criteria and data extraction has been ongoing since August 2022.

**Conclusion:**

In this systematic review, we will identify and consolidate information and evidence related to the use and effectiveness of existing NLP approaches and tools for automatically detecting ADEs from free text (discharge summaries, General Practitioner notes, social media, etc.). Our findings will improve the understanding of the current landscape of the use of NLP for extracting ADEs. It will lead to better anticipatory management of predisposing factors with the potential to improve outcomes considerably. Our results will also be valuable both to NLP researchers developing methods to extract ADEs and to translational/clinical researchers who use NLP for this purpose and in healthcare in general. For example, from our initial analysis of the studies, we can conclude that the majority of the proposed works are about the detection (extraction) of ADEs from text. An important portion of studies also focus on the binary classification of text (for highlighting if it includes or not ADEs). Different challenges related to the unbalanced dataset, abbreviations and acronyms but also to the lower results with rare ADEs were also mentioned by the studied papers.

## Introduction

### Background

ADEs are harmful events or undesired harmful effects resulting from medications or other methods of treatment ^1^. ADEs are often precipitated by a particular trigger event such as an infection, and precipitants are often the focus of reactive healthcare. However, the occurrence and severity of ADEs are significantly determined by the presence of predisposing factors, which are a complex interaction between morbidities, medicines/treatments, and other individual characteristics, including frailty and wider social support ^2, 3^. For example, in the United States, ADEs are affecting hundreds of thousands of people and costing billions of dollars in outpatient settings in the U.S. alone, with these costs showing an increasing trend. Detection of ADEs is one of the main tasks in the pharmaceutical industry, where monitoring drug side effects is a crucial task for pharmaceutical companies developing drugs and the Food and Drug Administration (FDA). These side effects can lead to potential health hazards that are costly, forcing a drug withdrawal from the market ^4, 5^.

Large numbers of ADEs are recorded in different sources (including discharge summaries, General Practitioner (GP) notes, electronic medical records and social and medical reviews) ^6^. Detecting ADEs from free text is a new research area attracting interest. Using NLP techniques to automatically extract ADEs from such unstructured textual information helps clinical experts to effectively and efficiently use them in daily practices ^7^. It is of significance to understand and synthesise recent developments specific to detecting ADEs using NLP as this will assist researchers in gaining a broader understanding of the field and provide insight into methods and techniques supporting and promoting new developments in the field.

As the number of approaches relying on NLP for extracting meaningful information (including ADEs) from medical text increased each year, it is critical to synthesise, classify and extract meaningful knowledge from research studies that have already been proposed. To this end, we conduct a systematic review to explore, organize, and understand the breadth of knowledge regarding the use of NLP for the detection/extraction of ADEs (which is referred to as a "detection" process throughout this study) ^8– 10^.

### Objectives

This systematic review has four major objectives: (1) identify the NLP applications used to predict ADEs; (2) highlight the techniques used for evaluating the proposed approaches and models; (3) identify the datasets used for training and fine-tuning the proposed models; (4) identify the key elements presented in the annotation schemas used for preparing the training data.

These objectives align with the systematic review methodology. Specifically, systematic reviews can serve as a valuable tool in sharing essential insights from the vast and intricate research literature with policymakers and practitioners who may not have sufficient time to scrutinize the credibility and dependability of individual studies. Additionally, these reviews provide an occasion for the research community to assess the calibre of the existing research and its reporting, thus avoiding unnecessary duplication of effort.

### Novelty

The use of NLP in medicine, in general, is a growing research area allowing the prediction of patient outcomes in critical care ^11– 14^. Locke et al.'s review ^11^. reveals that nearly all the examined works were published post-2017. Consequently, there are very few reviews on the use of NLP for detecting adverse events. Only three reviews on the detection of ADEs using NLP were identified ^15– 17^. The first one by Wong *et al.* ^15^ is a narrative review presenting an introduction to NLP applied to medication safety, along with a discussion of possible future directions and opportunities for applying NLP to enhance medication safety. The main strength of this paper is the review of the utility of NLP in four sources: Electronic Health Records (EHRs), Internet-based data, medical literature, and reporting systems. However, these authors only rely on the MEDLINE database. Also, their review considers the papers that have been published between 2007 and 2017 where the majority of NLP advances, are mainly related to the development of foundation models (including Bidirectional Encoder Representations from Transformers (BERT) ^18^, Generative Pre-Trained Transformers (GPT) ^19^ and Pathways Language Model (PaLM) ^20^) after 2017. Hence reviewing the literature from 2017 onward seemed crucial to us when we began our own systematic review.

The second review by Young *et al.* ^16^ presents a systematic review limited to studies evaluating NLP methods for the classification of incident reports and adverse events in healthcare. The main strength of this review is related to the diversity of libraries that have been queried (including Medline, Embase, The Cochrane Library, CINAHL, MIDIRS, ISI Web of Science, SciELO, Google Scholar, and PROSPERO). A grey literature search was also conducted via OpenGrey. The authors also relied on a large time window, including papers from 2004 to 2018. However, this review limited its search to studies proposing methods for the classification task only. Classification is only one of the tasks that can be applied to detect ADEs using NLP. Other tasks such as extraction (mainly using named entity recognition) or normalisation (known as entity linking) to a medical ontology are also being used. Hence, reviewing all these tasks and the methods used is crucial to gaining a comprehensive understanding of the research area.

The third publication by Murphy *et al.* ^17^ presents a scoping review dedicated to the detection of ADEs using NLP. Published in 2023, this is the most recent review in the domain to the best of our knowledge. It also focused on the most recent studies (published between 2011 and 2021). However, the search was performed on only three databases (Medline, Embase and arXiv). The authors also limited the datasets being considered by including studies applying their approaches to clinical narratives from EHRs only. Other data sources can provide a valuable source, for example, data from social media. Pharmaceutical companies have taken an interest in learning what people think and report about their products, which requires the application of NLP techniques to collect, extract, represent, analyse, and verify data (mainly related to ADEs) from social media such as Twitter, Reddit, Instagram, Facebook, forums, etc. Hence, focusing on different data sources is crucial to have a broad representation of the proposed studies.

To bridge the gap, we are proposing a systematic review focusing on the most recent studies that have been conducted on the detection of ADEs using NLP where all the extracted papers were published between 01/01/2017 and 12/31/2022. In order to have better coverage of the studies related to the detection of ADEs using NLP, we used six libraries in total (i.e. Embase, Medline, Web of Science, ACM Guide to Computing Literature, IEEE Digital Library and Scopus). We also defined (with the help of the University Academic Support Librarians) consistent search queries for each library allowing us to return the most relevant papers involving ADEs and NLP. We also relied on the Covidence tool for all the steps (both screening and data extraction), which assisted the reviewers in an interactive way. Finally, we conducted a pilot phase for each step of the process. During this phase, a sample of papers (two for the data extraction step) was extracted and assessed by the reviewers. The results were then discussed, which allowed us to improve the proposed protocol and refine the data extraction template.

## Methods

### Ethics approval

This systematic review does not involve human participants and, as such, does not require ethics approval. The study has been registered on Prospero (CRD42022330531).

### Design

 Our approach involves a systematic process of developing one or more research questions, searching academic databases, screening search results, and extracting data from relevant studies for collation and dissemination.

For assessing the studies of any formal risk of bias, we adopted the Prediction Model Risk of Bias Tool (PROBAST) ^21^ as a guide. We followed the same strategy used by Huang *et al.* ^22^ and only considered 16 questions from the 20 questions initially included in PROBAST. Finally, we followed the PRISMA guidelines to ensure transparency and consistency in reporting ^23^.

### Stage 1: Identifying the research questions

The following research questions were developed through an iterative process involving discussions with the research team, including clinicians and NLP experts. Four main questions were proposed:

- How are current NLP tools being applied to predict adverse drug events?
- How are these approaches and models evaluated?
- What are the available datasets used for predicting adverse drug events?
- What are the characteristics of the annotation guideline (schema) used?

### Stage 2: Identifying relevant studies

The search strategy was developed in consultation with domain and technical experts as well as Dr Bohee Lee, Systematic Review Tutor at the Academic Support Librarians (University of Edinburgh).

We conducted the search on 6 academic databases, including Embase, Medline, Web Of Science Core Collection, ACM Guide to Computing Literature, IEEE Digital Library and Scopus, to identify literature that describes the use of NLP to automatically detect ADEs. The major concepts that defined keywords were related to "NLP" and "ADEs". As Scopus and Web of Science Core Collection do not use subject headings, we decided to not use headings for all our searches to remain consistent. The results of the academic literature searches were imported into Covidence software (Covidence systematic review software, Veritas Health Innovation, Melbourne, Australia. Available here) for deduplication and screening. The search strategy for each database is presented in more detail in Appendix 1 (Appendix 1 can be found on figshare ^24^).

### Stage 3: Selecting studies

The third stage of the systematic review was the study selection, which included an initial title and abstract screening, followed by full-text screening.

### Inclusion and exclusion criteria

Studies were deemed eligible for inclusion if they presented and validated solutions specifically aimed at identifying ADEs through NLP techniques, and if these studies were published in academic publications. We were also interested in the datasets related to each study. They are crucial for training models, hence recording any lexicons or corpora that are freely available is an important added value of this systematic review. The training phase for machine learning methods requires an annotated dataset. For preparing such annotated datasets, the first step is to prepare an annotation guideline supporting the annotators’ task of understanding what needs to be annotated and resolving all ambiguities. Hence, we are also interested in papers presenting and discussing annotation guidelines. However, we excluded reviews, protocols patent papers and any papers that were not published via peer review. We also excluded papers that were not in English or those that were not reporting ADEs related to human subjects. We also excluded papers that had no relationship with NLP or ADEs and those proposing approaches that were not validated or those that were not focused on free text.

More rules for including or excluding papers are presented in Appendix 2 (Appendix 2 can be found on figshare ^24^). Moreover, some papers were also provided for the reviewers with an indication for including/ excluding them for both stages of title and abstract screening and full-text screening. More details regarding those papers are presented in Appendix 3 (Appendix 3 can be found on figshare ^24^).

### Title and abstract screening

Independent screening of the title and abstract of each article was performed by two reviewers based on the inclusion and exclusion criteria. Overall, three reviewers performed this task (FF, CL and SA), where FF screened all papers and both CL and SA screened half of them each. Hence some papers were screened by FF and CL and others by FF and SA. The agreement between the reviewers can be described as moderate (following the classification of McHugh ^25^). The agreement between FF and SA was 0.56 and the agreement between FF and CA was 0.51 (both representing Cohen’s kappa value). This agreement is considered moderate as it is within the range of 0.41 to 0.60. If both reviewers included an article, it underwent full-text screening. If they disagreed, the paper was screened a third time by IG for resolving conflicts.

### Full text screening

Independent screening of the full text of each article was performed by two reviewers based on the inclusion and exclusion criteria. Overall, three reviewers performed this task(JW, AG and RT), where JW screened all papers and both AG and RT screened half of them each. Hence some papers were screened by JW and RT and others by JW and AG. The agreement of full-text screening between the reviewers was also moderate, with a score of 0.41 for JW and AG and a score of 0.49 for JW and RT. As for the abstract screening, if both reviewers included an article, it underwent data extraction. If they disagreed, the paper was screened again by IG for resolving conflicts.

**Figure 1.**
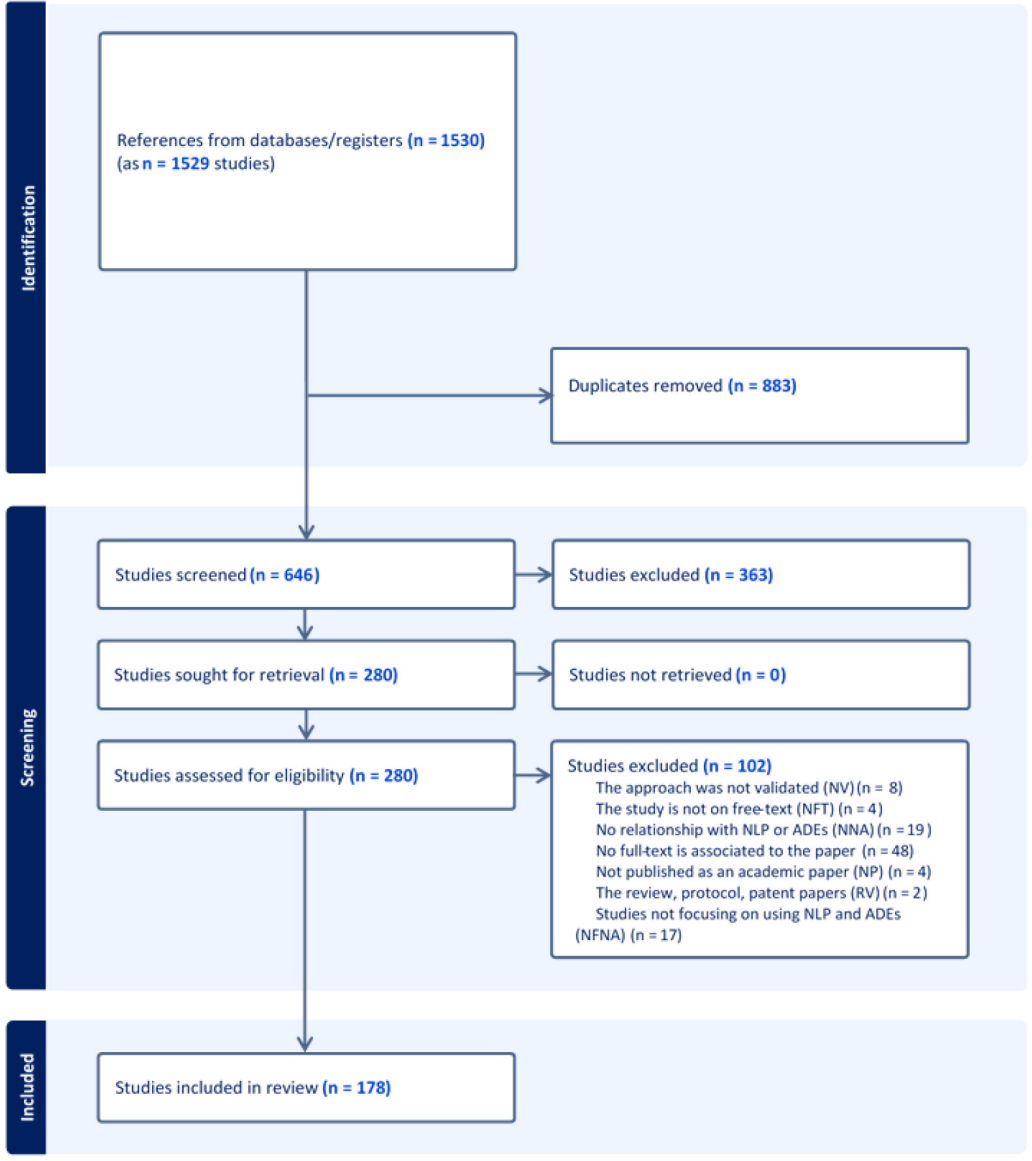
PRISMA diagram for search publication retrieval.

A PRISMA-ScR flowchart that outlines the search decision process and the number of studies included at each point of the process has been prepared ( Figure 1) and will be disseminated in the systematic review paper describing the completed review.

During this study, we use the Covidence tool for both screenings ^26^. Covidence also allows reviewers to resolve conflicts for papers where they did not agree. In the context of this study, the reviewers were asked to not resolve the conflicts, as this was the task of a third reviewer (IG).

### Stage 4: Charting the data

Data was independently extracted by four reviewers (JW, AG, YB, CN) from the articles included in the review and entered into a data charting form. All the data extracted is reviewed and validated by IG. The extraction phase is now complete with the data of 178 papers being extracted. The template for extracting the data was proposed on Covidence. Different kinds of data were extracted from each paper:

- **General information.** General information related to the title, type of publisher (conference, journal, etc.), name of publisher, year published, number of pages and the different institutions of the authors.
- **Data resources.** Data related to the resources that were used, created or mentioned. We are interested in the name of the resource, its type (i.e. corpus, lexicon, ontology, etc.), the type of data that has been used for constructing it (online health forums, discharge summaries, etc.), the source of the data ( Askpatient, WebMD, EHR, etc), the size of the resource (e.g. number of documents, sentences or words), the language of the resource, the link for accessing the resource (if publicly available) and finally the link to any licence for using the resource. All these types of information are collected for each dataset that was reported on.
- **Annotation information.** Information related to the type of annotation that has been done (manual, automatic, semi-automatic, etc.), the pre-processing that was done on the datasets and the tools that were used for pre-processing, the list of ADEs that were annotated and the list of the other tags that were used (such as conditions, drugs, etc), size of the dataset, number of documents including ADEs, link to the annotation guideline (if available), number of annotators, Inter-Rater Reliability (IRR) metrics that were used, the techniques for resolving conflicts that have been used, list of tools used for the automatic annotation (if applicable) as well as the number of documents annotated manually, automatically or semi-automatically (if applicable). We collected these types of information for each dataset that was annotated.
- **NLP tasks.** Tasks related to the main aim of the paper (ADEs classification, detection, normalisation/ linking, etc.). For each task, we extracted the approach that has been used (lexicon-based, corpus-based, etc.), the list of the resources (lexicons, corpora, ontology, etc.) used for training, validation or evaluation for each task and their size, the list of embedding models that were used for the task, the list of models and hyper-parameters that were used for each task.
- **Evaluation method.** Evaluation related to the techniques and the results (best and worst) from the evaluation of each task (precision, recall and F1-score). We are also interested in the comparison between each approach and the state-of-the-art (SOTA) approaches, hence if the results obtained in the SOTA are mentioned we are also recording them.
- **Demographic information.** The studied population related to the population characteristics that were considered for collecting the data. We are interested in information related to the age, gender, number of conditions related to the considered population (in the case of multi-morbidity), number of drugs taken (in the case of polypharmacy), and other information related to the population that is reported and may be of interest.
- **Other information.** Finally, we are also interested in extracting other information such as the list of drugs that were targeted (if applicable), the challenges that the authors faced, common ADEs that were detected, other interesting information reported (i.e. any reference to an interesting dataset, or link to the code of tools) that were not extracted earlier and how the paper could be improved (which is either mentioned in the discussion part or suggested by one of the reviewers).

The risk of bias and reporting quality were assessed using the PROBAST tool. PROBAST assesses the risk of bias for diagnostic and prognostic prediction model studies. It is organized into 4 domains (participants, predictors, outcome, and analysis) containing 20 signalling questions.

The evaluation process encompassed four key domains: participant selection methods and data sources or study designs were reviewed within the participant domain; predictor definitions and measurements were assessed for potential biases and applicability; outcomes were evaluated for study definitions and measurements that could be subject to bias and applicability concerns; and statistical analyses were scrutinized for feature extraction or text representation methods, algorithm selection, feature selection techniques, and hyperparameter tuning. Each domain was rated for risk of bias (ROB) using a predefined scale: “yes” (+), “probably yes,” “probably no,” “no” (−), and “no information,” where “yes” (+) indicated low risk and “no” (−) signified high risk. Studies were classified as high ROB if at least one domain was deemed high risk, low ROB if all domains were rated as low risk, and unclear ROB when the assessment was inconclusive.

Following ^22^, signalling questions 3.3, 4.2, 4.5, and 4.9 were not assessed in this study because they are not applicable to NLP. The predictor in NLP is free text, predictors are naturally excluded from the outcome definition (3.3). Item 4.2 was not assessed as text data are not by nature continuous or categorical; preprocessing of text to numeric data was assessed in item 4.6. Finally, univariable analysis is not applicable to text data (4.5), and assigning weights to the predictors does not apply either (4.9). The same reviewers (JW, AG, YB, CN) extracting the data were involved in the quality assessment process (and it was validated by IG).

### Stage 5: Collating and reporting the results

Characteristics and findings from all included literature will be tabulated and summarized, and aggregated data will be presented. We will conduct a manual thematic analysis of the included studies to highlight key themes emerging from the literature. We also plan to summarise data using meta-analysis because of the anticipated heterogeneous study design, objectives, NLP techniques, and reported outcomes. We plan to conduct a narrative synthesis for the included studies. We will summarize the studies using the same attributes for each study in the same order. We will also provide a narrative summary of each subgroup according to the data characteristics and to the study design. We will report both a qualitative summary and quantitative analysis where possible.

The reviewed publications will be grouped in tables with respect to their tools, models and approaches used. A further summary table will be dedicated to the classification of the data used regarding the annotation technique, the tool used for annotation, any guidelines provided, and the size of the different corpora used for training, validation and testing. We also plan to provide statistics related to the occurrence of ADEs in the studied population. Finally, we will illustrate the most common ADEs by population and/or by disease in histograms and pie charts. We are currently within this stage, where we are analysing and reporting the outcomes from this systematic review.

### Stage 6: Patient and Public Involvement

This systematic review was co-designed by a multidisciplinary team using an integrated knowledge translation approach. Stakeholders and knowledge users, including clinicians, computer scientists and NLP experts are contributing to all stages of the study. Team members assisted in developing the research question, defining the scope of the search strategy, and identifying relevant data extraction elements. They also assisted in developing a methodology for searching the literature. Some members of the public have also been involved in our work on ADE in general. They were solicited to prepare a list of the most frequent ADEs that they might experience. A Public and Patient Involvement and Engagement (PPIE) event was also organised on August 31st, 2022 which was partly dedicated to the detection of ADEs using NLP and where the topic was discussed with the participants. During the event, the participants were informed of the use of NLP for detecting ADEs via a set of presentations. They were able to interact with our team of NLP experts/clinicians. They also participated in a group discussion to share their opinion and thoughts regarding the ADEs that they might experience, a classification of the severity of these events and their sentiment regarding sharing their medical data for research purposes.

This event was not only beneficial for the preparation of this systematic review but also for our other research on the detection of side effects using NLP. There was a lot of discussion about how to know that a symptom is a side effect of medication and not a symptom of a condition in itself, and particularly how a connection could be made using medical records/text unless explicitly stated in the written text. Attendees mentioned starting and stopping medications to ’test’ what possible drug interactions or side effects they may be experiencing. Attendees also mentioned that some side effects of medications can be compounded when taking multiple medications together. For example, many medications can cause constipation so when taking them together this becomes a major issue. The challenges of polypharmacy and the feeling of having to self-manage multiple conditions came up repeatedly throughout the day, and there was a general consensus that side effects of medications create anxiety when living with long-term conditions.

## Study status

As of December 2024, we have completed stages 1, 2 and 3 of the systematic review. We identified 178 studies for inclusion through the academic literature search ^21^. Currently, we are writing the paper (stage 5). Further refinement of the eligibility criteria and data extraction has been ongoing since August 2022 (stage 4). Dissemination are expected to occur on February 2025.

## Discussion

### Overview

In this systematic review, we will identify and consolidate information and evidence related to the use of existing NLP approaches and tools for detecting ADEs from free text (discharge summaries, social media data, etc.). Based on the preliminary results of this review, we hypothesize that our findings will demonstrate heterogeneity in the types and diversity of approaches and tools being used. Additionally, this systematic review will lay a foundation for exploring the effective evaluation of tools as part of future research. It will also record and examine the datasets and the annotation guidelines that have been constructed and proposed for developing and training different models.

### Limitations

The development of this protocol for our review serves to provide a detailed structure for the systematic review to improve the transparency of the research. However, our study presents some limitations. We focused on papers that have been published in academic journals and conferences. Hence, we may be missing some studies that are in the process of publication that were disseminated in a given repository (such as ArXiv) but had not completed peer review at the time of data collection. This research was done using the library access of the University of Edinburgh granting us access to the majority of published papers. However, we were not able to find and obtain the full text of 48 papers. Hence, those papers were excluded from our review. Also, the protocol was prepared even before collecting the papers and was improved after the collection. Hence, we exclude some papers on drug-drug interactions which do not explicitly fall within the scope. Moreover, because we had to extract an important quantity of data from 178 papers, the dissemination of this paper has taken more time than initially expected.

Finally, the search strategy was implemented across six academic databases, selected in collaboration with domain and technical experts as well as a Systematic Review Tutor from the Academic Support Librarians at the University of Edinburgh. This approach facilitated the identification of a substantial body of relevant literature, resulting in the inclusion of 178 studies in the systematic review. While every effort was made to ensure comprehensive coverage, it is acknowledged that no review can encompass all possible databases. Based on feedback received during the review process, we recognize that including the ACL family of libraries could have further enriched the scope of this review. Our synthesis identified several studies published in ACL venues, highlighting the importance of integrating such libraries to capture a broader spectrum of relevant research. This insight informs recommendations for future systematic reviews in this area.

### Dissemination plan

The findings from this systematic review will firstly be presented internally to an interdisciplinary team working on Artificial Intelligence and Multimorbidity: Clustering in Individuals, Space and Clinical Context (AIM-CISC) at the University of Edinburgh and will then be shared in the wider UK AIM network and with international collaborators. The outputs are also of interest to ACRC, the Advanced Care Research Centre, a multi-disciplinary research program combining research across fields including medicine and other care professions, engineering, informatics, data and social sciences. We also plan to disseminate the results to the members of the PPIE group that participated in our event in August 2022. Finally, we plan to publish this systematic review in an international journal on health informatics or in a biomedical journal.

## Data Availability

No data are associated with this article. Figshare: Appendix/Prisma P, https://doi.org/10.6084/m9.figshare.24541843.v1 ^24^. This project contains the following extended data: Appendix.pdf Figshare: Prisma P checklist for ’Natural Language Processing for Detecting Adverse Drug Events: Systematic Review Protocol’, https://doi.org/10.6084/m9.figshare.24541843.v1 ^24^.

## References

[1] Rawal S Rawal S Anwar S : Identification of adverse drug reaction mentions in tweets-smm4h shared task 2019. In: *Proceedings of the Fourth Social Media Mining for Health Applications (# SMM4H) Workshop & Shared Task*.2019;136–137. Reference Source

[2] Wu H Toti G Morley KI : Semehr: a general-purpose semantic search system to surface semantic data from clinical notes for tailored care, trial recruitment, and clinical research. *J Am Med Inform Assoc.* 2018;25(5):530–537. 10.1093/jamia/ocx160 29361077 PMC6019046

[3] Kharrazi H Anzaldi LJ Hernandez L : The value of unstructured electronic health record data in geriatric syndrome case identification. *J Am Geriatr Soc.* 2018;66(8):1499–1507. 10.1111/jgs.15411 29972595

[4] Yazdani A Rouhizadeh H Bornet A : CONORM: context-aware entity normalization for Adverse Drug Event detection. *medRxiv.* 2023; 2023-09. 10.1101/2023.09.26.23296150

[5] Hsu D Moh M Moh TS : Drug side effect frequency mining over a large twitter dataset using apache spark. In: *Handbook of Artificial Intelligence in Biomedical Engineering. *Apple Academic Press,2021;233–259. Reference Source

[6] Ding X Mower J Subramanian D : Augmenting aer2vec: enriching distributed representations of adverse event report data with orthographic and lexical information. *J Biomed Inform.* 2021;119: 103833. 10.1016/j.jbi.2021.103833 34111555 PMC8260467

[7] Shen Z Spruit M : Automatic extraction of adverse drug reactions from summary of product characteristics. *Appl Sci.* 2021;11(6): 2663. 10.3390/app11062663

[8] Binkheder S Wu HY Quinney SK : PhenoDEF: a corpus for annotating sentences with information of phenotype definitions in biomedical literature. *J Biomed Semantics.* 2022;13(1): 17. 10.1186/s13326-022-00272-6 35690873 PMC9188713

[9] Combi C Zorzi M Pozzani G : Normalizing spontaneous reports into MedDRA: some experiments with magiCoder. *IEEE J Biomed Health Inform.* 2019;23(1):95–102. 10.1109/JBHI.2018.2861213 30059326

[10] Yang X Bian J Gong Y : MADEx: a system for detecting medications, adverse drug events, and their relations from clinical notes. *Drug Saf.* 2019;42(1):123–133. 10.1007/s40264-018-0761-0 30600484 PMC6402874

[11] Locke S Bashall A Al-Adely S : Natural language processing in medicine: a review. *Trends Anaesth Crit Care.* 2021;38:4–9. 10.1016/j.tacc.2021.02.007

[12] van Aken B Papaioannou JM Mayrdorfer M : Clinical outcome prediction from admission notes using self-supervised knowledge integration. In: *Proceedings of the 16th Conference of the European Chapter of the Association for Computational Linguistics: Main Volume.*Association for Computational Linguistics,2021;881–893. 10.18653/v1/2021.eacl-main.75

[13] Naik A Parasa S Feldman S : Literature-augmented clinical outcome prediction. In: *Findings of the Association for Computational Linguistics: NAACL 2022*. Seattle, United States, Association for Computational Linguistics,2022;438–453. 10.18653/v1/2022.findings-naacl.33

[14] Gema A Daines L Minervini P : Parameter-efficient fine-tuning of Llama for the clinical domain. *arXiv preprint.* arXiv: 2307.03042.2023. 10.48550/arXiv.2307.03042

[15] Wong A Plasek JM Montecalvo SP : Natural language processing and its implications for the future of medication safety: a narrative review of recent advances and challenges. *Pharmacotherapy.* 2018;38(8):822–841. 10.1002/phar.2151 29884988

[16] Young IJB Luz S Lone N : A systematic review of natural language processing for classification tasks in the field of incident reporting and adverse event analysis. *Int J Med Inform.* 2019;132: 103971. 10.1016/j.ijmedinf.2019.103971 31630063

[17] Murphy RM Klopotowska JE de Keizer NF : Adverse drug event detection using natural language processing: a scoping review of supervised learning methods. *PLoS One.* 2023;18(1): e0279842. 10.1371/journal.pone.0279842 36595517 PMC9810201

[18] Devlin J Chang MW Lee K : BERT: Pre-training of deep bidirectional transformers for language understanding. In: *Proceedings of the 2019 Conference of the North American Chapter of the Association for Computational Linguistics: Human Language Technologies, Volume 1 (Long and Short Papers).*Minneapolis, Minnesota, Association for Computational Linguistics.2019;4171–4186. Reference Source

[19] Radford A Narasimhan K Salimans T : Improving language understanding by generative pre-training.2018. Reference Source

[20] Chowdhery A Narang S Devlin J : Palm: scaling language modeling with pathways. *arXiv preprint.* arXiv: 2204.02311.2022. Reference Source

[21] Wolff RF Moons KGM Riley RD : Probast: a tool to assess the risk of bias and applicability of prediction model studies. *Ann Intern Med.* 2019;170(1):51–58. 10.7326/M18-1376 30596875

[22] Huang BB Huang J Swong KN : Natural language processing in spine surgery: a systematic review of applications, bias, and reporting transparency. *World Neurosurg.* 2022;167:156–164. e6. 10.1016/j.wneu.2022.08.109 36049723

[23] Moher D Liberati A Tetzlaff J : Preferred reporting items for systematic reviews and meta-analyses: the PRISMA statement. *Int J Surg.* 2010;8(5):336–341. 10.1016/j.ijsu.2010.02.007 20171303

[24] Guellil I Wu J Gema P : Appendix/Prisma P. *figshare.* Journal contribution.2023. 10.6084/m9.figshare.24541843.v1

[25] McHugh ML : Interrater reliability: the kappa statistic. *Biochem Med (Zagreb).* 2012;22(3):276–282. 23092060 PMC3900052

[26] Babineau J : Product review: covidence (systematic review software). *JCHLA / JABSC.* 2014;35(2):68–71. 10.5596/c14-016

[27] Casey A Davidson E Poon M : A systematic review of natural language processing applied to radiology reports. *BMC Med Inform Decis Mak.* 2021;21(1): 179. 10.1186/s12911-021-01533-7 34082729 PMC8176715

