# Supplementary material for: Natural language processing for detecting adverse drug events: A systematic review protocol
Source: NIHR Open Res. 2024 Dec 10;3:67. Originally published 2023 Dec 19. [Version 3] doi: 10.3310/nihropenres.13504.3 (PMC11808655; doi:10.3310/nihropenres.13504.3)
Supplement: Peer review history

# Reviewer report: Reviewer response for version 3

**DOI:** 10.3310/nihropenres.15043.r34031

**Published:** 2024-12-12

**Authors:** Shuntaro Yada

**Affiliations:** 1 Nara Institute of Science and Technology, Nara, Japan; 2 Faculty of Library, Information and Media Science, University of Tsukuba, Tsukuba, Ibaraki Prefecture, Japan

Finally, the authors clarified why and how ACL papers are included in this time of systematic review. The decision seems reasonable, and the future direction and limitations sound decent.

Is the study design appropriate for the research question?

Yes

Is the rationale for, and objectives of, the study clearly described?

Yes

Are sufficient details of the methods provided to allow replication by others?

Yes

Are the datasets clearly presented in a useable and accessible format?

Not applicable

Reviewer Expertise:

Natural language processing and its application to medical and clinical domains.

I confirm that I have read this submission and believe that I have an appropriate level of expertise to confirm that it is of an acceptable scientific standard.

# Reviewer report: Reviewer response for version 2

**DOI:** 10.3310/nihropenres.14851.r32762

**Published:** 2024-09-10

**Authors:** Belén Otero Carrasco

**Affiliations:** 1 Polytechnic University of Madrid, Madrid, Community of Madrid, Spain

The paper describes a topic of great scientific interest and the objectives to be met and implemented are of great interest. However, throughout the article, it is not clear whether the proposed objectives have been met. It is true that, as the main result, the number of articles that have been selected to obtain the ADEs is indicated, but it is not indicated whether the relevant information could be extracted from these articles as proposed as an objective. 

 In the abstract, the information provided is not relevant to understand the research, for example, in the results section include this sentence “we are writing up the systematic review paper..." it is not of interest that a paper is being written and the number of ADEs detected in the 178 papers selected would be. This whole section in general needs to be revised for a better understanding of the research. 

 I consider that more emphasis should be placed on the proposed method and not on what has been previously indexed. It is also striking that there are only 3 articles that talk about this topic when NLP techniques are constantly used in health data. Some extra references found are included: *Identifying adverse drug reactions from patient reviews on social media using natural language processing* https://doi.org/10.1177/14604582221136712 , *A Natural Language Processing Framework for Detecting Adverse Drug Reactions in Clinical Structured Drug Reviews *doi: 10.34172/ijdrc.2024.e9

 On the other hand, it is considered that a more detailed explanation of the PROBAST method is missing, it is mentioned in different parts of the article but its functionality, why this method has been selected and the advantages it has over others is not clear. 

 As for the discussion,more extensive and detailed information on the contributions highlighted in this article is lacking

 At a general level, it is expected an improvement in the wording of the article that will help to better understand all the detailed processes.

Is the study design appropriate for the research question?

Partly

Is the rationale for, and objectives of, the study clearly described?

Partly

Are sufficient details of the methods provided to allow replication by others?

Partly

Are the datasets clearly presented in a useable and accessible format?

Not applicable

Reviewer Expertise:

Computational biology, Artificial Intelligence and data-driven approaches

I confirm that I have read this submission and believe that I have an appropriate level of expertise to confirm that it is of an acceptable scientific standard, however I have significant reservations, as outlined above.

## References

[1] : Identifying adverse drug reactions from patient reviews on social media using natural language processing. *Health Informatics J* .2023;29(1) : 10.1177/14604582221136712 14604582221136712 10.1177/14604582221136712 36857033

[2] : Developing a deep learning natural language processing algorithm for automated reporting of adverse drug reactions. *J Biomed Inform* .2023;137: 10.1016/j.jbi.2022.104265 104265 10.1016/j.jbi.2022.104265 36464227

# Response

**Published:** 2024-12-05

**Authors:** Imane Guellil

**Affiliations:** The University of Edinburgh, Edinburgh, Scotland, UK

We would like to thank the reviewer for acknowledging the scientific value of our paper. However, we believe there may be some misunderstanding regarding the type of paper being presented. This submission is a systematic review protocol, not the systematic review itself. The purpose of this protocol is to describe the methodology and steps undertaken to propose and plan the systematic review, in accordance with PRISMA guidelines. This protocol is a necessary step prior to conducting the review, which ensures transparency and rigour in the systematic review process. The systematic review, which we are currently drafting, will present and discuss the results of the 178 studies identified through the process outlined in this protocol.

 A systematic review protocol aims to provide a detailed outline of how the systematic review will be conducted. It includes defining the research questions, specifying the databases and libraries used, and describing the screening and selection processes for studies. This is precisely the focus of the present paper. To provide clarity, we have also included examples of similar research protocols that follow a structure comparable to ours. The results and synthesis of the 178 studies will be thoroughly addressed in the forthcoming systematic review paper.

 We would also like to clarify that the reference to "three articles" pertains to the number of systematic reviews similar to ours, not the number of studies conducted on the use of NLP for detecting adverse events. Our systematic review process, as described in the protocol, led to the identification of 178 studies after completing both screening phases. These studies were synthesized, and their findings will be presented in detail in the systematic review paper following this protocol.

 We agree with the reviewer’s suggestion that some sections of the protocol require further elaboration, particularly regarding the PROBAST tool. We will revise the manuscript to incorporate these clarifications and provide additional details. Once again, we extend our gratitude to the reviewer for their time and constructive feedback, which will greatly enhance the quality of our paper.

https://pmc.ncbi.nlm.nih.gov/articles/PMC10729196/

https://journals.plos.org/plosone/article?id=10.1371/journal.pone.0259499

https://www.medrxiv.org/content/10.1101/2024.07.02.24309815v1.full.pdf

https://www.mdpi.com/1660-4601/19/19/12038

# Reviewer report: Reviewer response for version 2

**DOI:** 10.3310/nihropenres.14851.r32458

**Published:** 2024-07-27

**Authors:** Shuntaro Yada

**Affiliations:** 1 Nara Institute of Science and Technology, Nara, Japan; 2 Faculty of Library, Information and Media Science, University of Tsukuba, Tsukuba, Ibaraki Prefecture, Japan

My major point is not addressed well.

 It is unclear how the current database choice covers the ACL conferences, where the NLP studies are primarily made.

 For instance, the ACM database only includes some proceedings of the old (pre-2010 or so) ACL-family conferences; other databases seem not to include ACLs and related conferences and workshops at all.

 I could not check some commercial databases due to the lack of access, so some may include them but I do not tell it.

 Thus, the authors must clarify why and how these data sources are enough for the present systematic review.

Is the study design appropriate for the research question?

Yes

Is the rationale for, and objectives of, the study clearly described?

Yes

Are sufficient details of the methods provided to allow replication by others?

Yes

Are the datasets clearly presented in a useable and accessible format?

Not applicable

Reviewer Expertise:

Natural language processing and its application to medical and clinical domains.

I confirm that I have read this submission and believe that I have an appropriate level of expertise to confirm that it is of an acceptable scientific standard, however I have significant reservations, as outlined above.

# Response

**Published:** 2024-12-05

**Authors:** Imane Guellil

**Affiliations:** The University of Edinburgh, Edinburgh, Scotland, UK

We sincerely thank the reviewer for their insightful and valuable comments. To address the concern raised, we would like to clarify that the search strategy and the selection of databases were determined in consultation with domain and technical experts, as well as a systematic review tutor. Additionally, as noted in our previous response, our approach aligns with similar systematic reviews in the field, which also do not include the ACL family libraries within their database selections.

 Conducting a systematic review necessitates making informed choices, including the selection of databases, which we based on domain relevance, prior systematic reviews, and expert recommendations. We believe that the libraries we targeted were sufficient to capture the most pertinent studies in our area of focus. Furthermore, we observed that many of the screened papers included research from the ACL family of venues, even though the libraries were not specifically targeted.

 That said, we fully acknowledge the reviewer's point and agree that the absence of the ACL family libraries is a limitation of our work. We have now explicitly noted this limitation in the **Limitations** section of our manuscript. Additionally, as we proceed with writing the systematic review paper associated with this research protocol, we will ensure that this limitation is clearly acknowledged there as well.

 Once again, we express our gratitude to the reviewer for their constructive feedback, which has greatly contributed to improving the quality of our work.

 This part is added to the paper: "The search strategy was implemented across six academic databases, selected in collaboration with domain and technical experts as well as a Systematic Review Tutor from the Academic Support Librarians at the University of Edinburgh. This approach facilitated the identification of a substantial body of relevant literature, resulting in the inclusion of 178 studies in the systematic review. While every effort was made to ensure comprehensive coverage, it is acknowledged that no review can encompass all possible databases. Based on feedback received during the review process, we recognize that including the ACL family of libraries could have further enriched the scope of this review. Our synthesis identified several studies published in ACL venues, highlighting the importance of integrating such libraries to capture a broader spectrum of relevant research. This insight informs recommendations for future systematic reviews in this area."

# Reviewer report: Reviewer response for version 1

**DOI:** 10.3310/nihropenres.14654.r31380

**Published:** 2024-04-19

**Authors:** Braja Gopal Patra

**Affiliations:** 1 Cornell University, Ithaca, New York, USA

This manuscript discusses the NLP development on extracting adverse drug events. This could be an interesting task for researchers in this domain. However, there is not much information available in this manuscript. Here are my feedback:

 Pros:

 1. The authors did a great job in collecting the publications. Going through 1500 publications is not an easy task.  

 2. The inclusion of manuscripts from different categories of text (EHR, social media) is a good one. 

 3. Several literature review techniques (PRISMA, PROBAST) were used. 

 Cons:

 1. In abstract: The abstract provides nothing in the result section except the number of papers. The authors can focus on what are the NLP methods used or the type of data used. 

 2. Writing can be significantly improved. Missing bracket, NLP and ADEs abbreviations are described on multiple places (in introduction, Methods Stage 3).

 3. The authors discussed about annotation guideline in Methods (stage 3). How did annotation guideline help in including or excluding manuscripts?

Is the study design appropriate for the research question?

No

Is the rationale for, and objectives of, the study clearly described?

Yes

Are sufficient details of the methods provided to allow replication by others?

No

Are the datasets clearly presented in a useable and accessible format?

Not applicable

Reviewer Expertise:

Natural Language  Processing, Social Determinants of Health, Electronics Health Records

I confirm that I have read this submission and believe that I have an appropriate level of expertise to confirm that it is of an acceptable scientific standard, however I have significant reservations, as outlined above.

# Response

**Published:** 2024-07-18

**Authors:** Imane Guellil

**Affiliations:** The University of Edinburgh, Edinburgh, Scotland, UK

Dear reviewer,

 Thank you very for your useful comments.

 We take all of them into consideration.

 Please see below our detailed answer to each one of them:

 1) In abstract: The abstract provides nothing in the result section except the number of papers. The authors can focus on what are the NLP methods used or the type of data used. --> **This short paragraph was added at the end of the abstract: “For example, from our initial analysis of the studies, we can conclude that the majority of the proposed works are about the detection (extraction) of ADEs from text. An important portion of studies also focus on the binary classification of text (for highlighting if it includes or not ADEs). Different challenges related to the unbalanced dataset, abbreviations and acronyms but also to the lower results with rare ADEs were also mentioned by the studied papers.”. Please note that we can also enrich this part with other NLP conclusions, if needed.**

 2) Writing can be significantly improved. Missing bracket, NLP and ADEs abbreviations are described on multiple places (in introduction, Methods Stage 3). --> **Done. The writing was improved and all the duplicated mentions of NLP and ADE abbreviations were removed.**

 3) The authors discussed about annotation guideline in Methods (stage 3). How did annotation guideline help in including or excluding manuscripts? --> **The annotation guideline mentioned is not for including/excluding manuscripts. However, it is one data we are interested in extracting from the different studied manuscripts. As the proposed papers are about the use of NLP for detecting ADEs, some papers are about the construction of resources (dataset). For annotating such a dataset, an annotation guideline is required. Hence, we are also interested in the information presented about these annotation guidelines (as only a few presented in the research literature).**

# Reviewer report: Reviewer response for version 1

**DOI:** 10.3310/nihropenres.14654.r31155

**Published:** 2024-04-17

**Authors:** SU Golder

**Affiliations:** 1 University of York, Heslington York, UK

This is a very important topic - the detection of ADEs from unstructured data. Consideration of ADEs is important for informed decision-making, be it by policy makers, clinicians or patients. Currently not enough emphasis is placed on ADE in evidence-based medicine. Detecting ADEs, however, can be challenging. There are many different sources that can be useful but ADEs are often considered secondary or even tertiary outcomes and often 'hidden'. Useful data on ADEs can be found in unstructured data but unstructured data by its very nature can be difficult to search. It is this particular challenge that the systematic review that the authors plan to undertake will help to address with their four objectives of identifying NLP applications, highlighting techniques for evaluating the approaches, identifying datasets, and key elements in annotation schemas. I very much look forward to reading the completed systematic review.

 Major points: The article needs more clarity. The tense used as to what has been done and what will be done is inconsistent with the statement on their progress. They also mention following scoping review guidance but they are doing a systematic review. The searches were conducted in 2022 so in such a fast moving area I am concerned about the time lag to completion and publication of the systematic review itself. 

 Minor points: Web of Science is an interface not a database so be good if the authors could list the databases searched within Web of Science. The fields within which each term is searched are not recorded in the appendix, please add. Abbreviations are not always in full on first occurrence. It is unclear what is meant by 'plausible interventions' in the introduction, please clarify.  I also think the importance of detecting ADEs and the importance of using unstructured data could be more strongly emphasized. Some sections throughout the protocol that would benefit from references (particularly in the introduction). The three sentences in 'Novelty' beginning with The review by Locke - would be better reworded for clarity.

Is the study design appropriate for the research question?

Yes

Is the rationale for, and objectives of, the study clearly described?

Yes

Are sufficient details of the methods provided to allow replication by others?

Partly

Are the datasets clearly presented in a useable and accessible format?

Yes

Reviewer Expertise:

Systematic reviews, Adverse events, Social media

I confirm that I have read this submission and believe that I have an appropriate level of expertise to confirm that it is of an acceptable scientific standard, however I have significant reservations, as outlined above.

# Response

**Published:** 2024-07-18

**Authors:** Imane Guellil

**Affiliations:** The University of Edinburgh, Edinburgh, Scotland, UK

Dear reviewer,

 Thank you very for your useful comments.

 We take all of them into consideration.

 Please see below our detailed answer to each one of them:

 1) The article needs more clarity. The tense used as to what has been done and what will be done is inconsistent with the statement on their progress. --> **All the stages (3 stages of the systematic review) are now done. We are currently writing up the paper. We update this on the protocol. We also make it clearer by adding the following statement:**

1. **In the abstract: Right now, we are writing up the systematic review paper where we are synthesising different findings.**
2. **In Stage 4: The extraction phase is now complete with the data of 178 papers being extracted. **
3. **In stage 4: We are currently within this stage, where we are analysing and reporting the outcomes from this systematic review.**

**2) **They also mention following scoping review guidance but they are doing a systematic review.  --> **The part referencing a scoping review was removed.**

**3) **The searches were conducted in 2022 so in such a fast moving area I am concerned about the time lag to completion and publication of the systematic review itself. --> **We are currently writing up the systematic review which will be submitted during these 2 months.**

**4) **Web of Science is an interface not a database so be good if the authors could list the databases searched within Web of Science --> **It was the Web Of Science Core Collection. This was updated on the protocol.**

**5) **The fields within which each term is searched are not recorded in the appendix, please add. --> **All the queries were performed on the title and abstract. This information is mentioned in appendix 1 related to the search strategy.**

**6) **Abbreviations are not always in full on first occurrence. --> **The paper was entirely reviewed and this was fixed. For example, abbreviations such as BERT or GPT are now associated with their full meaning.**

**7)** It is unclear what is meant by 'plausible interventions' in the introduction, please clarify. --> **This sentence was removed and replaced with another paragraph to better emphasise the importance of detecting ADEs.**

**8) **I also think the importance of detecting ADEs and the importance of using unstructured data could be more strongly emphasized --> **This paragraph was added: “For example, in the United States, ADEs are affecting hundreds of thousands of people and costing billions of dollars in outpatient settings in the U.S. alone, with these costs showing an increasing trend. Detection of ADEs is one of the main tasks in the pharmaceutical industry, where monitoring drug side effects is a crucial task for pharmaceutical companies developing drugs and the Food and Drug Administration (FDA). These side effects can lead to potential health hazards that are costly, forcing a drug withdrawal from the market**

 9) Some sections throughout the protocol that would benefit from references (particularly in the introduction). --> **Different references were added to the protocol including the introduction**

**10) **The three sentences in 'Novelty' beginning with The review by Locke --> **would be better reworded for clarity. -->These sentences were reformulated to: Locke et al.'s review reveals that nearly all the examined works were published post-2017. Consequently, there are very few reviews on the use of NLP for detecting adverse events. Only three reviews on the detection of ADEs using NLP were identified.**

# Reviewer report: Reviewer response for version 1

**DOI:** 10.3310/nihropenres.14654.r31041

**Published:** 2024-04-17

**Authors:** Shuntaro Yada

**Affiliations:** 1 Nara Institute of Science and Technology, Nara, Japan; 2 Faculty of Library, Information and Media Science, University of Tsukuba, Tsukuba, Ibaraki Prefecture, Japan

This paper proposes a protocol for a systematic review of adverse drug event detection. The protocol includes Embase, Medline, Web Of Science, ACM Guide to Computing Literature, IEEE Digital Library, and Scopus. The screening criteria moderately worked because the inter-reviewer agreement was around 0.4-0.6. This review finally identified 178 articles, and the paper review is still ongoing. The rest of the protocol details look solid and rigorous to me.

 I have one major comment and several minor comments.

 Major: Why do the target digital libraries not include ACL Anthology?

 ACL and family conferences and workshops are the central place for NLP studies, which definitely include adverse drug event detection (see BioNLP and SMM4H workshops). How can this NLP review be justified without including papers therein? I wonder why this systematic review is biased to journal papers (that are not necessarily focusing on NLP and are often outdated) by selecting the databases listed above, though the authors care about the recency of literature review in this field (as in the "Novelty" subsection). From this point of view, I am not satisfied with what the authors state in the "Limitation" subsection. Please clarify if the selected DBs include ACL papers. Otherwise, I believe this is the critical weakness of this paper.

 Minor:

 1) In the "Novelty" subsection, the authors compare the present study with the prior ones. I think the statement below regarding Wong et al. does not sound fair: "However, records were searched from the MEDLINE database only for potential studies from 2007 to 2017. This will drastically limit the number of collected papers." Because the present study's publication period is shorter than Wong et al.'s, though I reckon that the coverage of the DBs is larger than Wong et al.'s, I suggest that the authors make these two sentences sound fairer.

 2) Also, in the "Novelty" subsection, the last two sentences (starting with "Finally, we also ran...") are hard to understand. Could you please refine the writing?

 3) References contain many errors, such as wrong cases of titles.

Is the study design appropriate for the research question?

Yes

Is the rationale for, and objectives of, the study clearly described?

Yes

Are sufficient details of the methods provided to allow replication by others?

Yes

Are the datasets clearly presented in a useable and accessible format?

Not applicable

Reviewer Expertise:

Natural language processing and its application to medical and clinical domains.

I confirm that I have read this submission and believe that I have an appropriate level of expertise to confirm that it is of an acceptable scientific standard, however I have significant reservations, as outlined above.

# Response

**Published:** 2024-07-18

**Authors:** Imane Guellil

**Affiliations:** The University of Edinburgh, Edinburgh, Scotland, UK

Dear reviewer,

 Thank you very for your useful comments.

 We take all of them into consideration.

 Please see below our detailed answer to each one of them: 

 1) Why do the target digital libraries not include ACL Anthology. ACL and family conferences and workshops are the central place for NLP studies, which definitely include adverse drug event detection (see BioNLP and SMM4H workshops). How can this NLP review be justified without including papers therein **--> Our search strategy allows us to have 10 papers from ACL. 4 were relevant and will be included in the systematic review and 6 were irrelevant. Also, the selection of libraries was done in collaboration with our librarian at the University of Edinburgh who suggested using this list of libraries based on their experience with previous systematic review. Finally, we also observe that the majority of the most recent systematic reviews on clinical NLP do not use ACL within the list of their used libraries (Natural language processing for clinical notes in dentistry: A systematic review, Extracting cancer concepts from clinical notes using natural language processing: a systematic review, De-identification of clinical free text using natural language processing: A systematic review of current approaches, Applications of Natural Language Processing in Cardiology using Text Clinical Data: A Systematic Review, A systematic review on natural language processing systems for eligibility pre-screening in clinical research, A Systematic Review of NLP Applications in Clinical Healthcare: Advancement and Challenges,  Natural Language Processing of Clinical Notes on Chronic Diseases: Systematic Review, etc). However, we will consider to use ACL for our future reviews.**

 2)  I wonder why this systematic review is biased to journal papers (that are not necessarily focusing on NLP and are often outdated) by selecting the databases listed above, though the authors care about the recency of literature review in this field (as in the "Novelty" subsection) --> **This was just a mistake in the limitation section, that was corrected now. We did not focus only on the journals but also on conferences. However, we excluded any paper that has not been peer-reviewed and put on ArXiv. Also, we observe that our search strategy allows us to have papers from the Journal of Biomedical Semantics,  Studies in Health Technology and Informatics, IEEE Journal of Biomedical and Health Informatics, Bionlp and CEUR workshops but also ACL,  EACL and ACL-IJCNLP confs.**

 3) From this point of view, I am not satisfied with what the authors state in the "Limitation" subsection. Please clarify if the selected DBs include ACL papers. Otherwise, I believe this is the critical weakness of this paper. --> **As stated before, mentioning in the limitations section that we focus just on journals was a mistake and we corrected it. We also focus on conference papers and we got many conference and workshop papers using our search strategy.**

 4) In the "Novelty" subsection, the authors compare the present study with the prior ones. I think the statement below regarding Wong et al. does not sound fair: "However, records were searched from the MEDLINE database only for potential studies from 2007 to 2017. This will drastically limit the number of collected papers." Because the present study's publication period is shorter than Wong et al.'s, though I reckon that the coverage of the DBs is larger than Wong et al.'s, I suggest that the authors make these two sentences sound fairer --> **These sentences were replaced by: However, these authors only rely on the MEDLINE database. Also, their review considers the papers that have been published between 2007 and 2017 where the majority of NLP advances, are mainly related to the development of foundation models (including Bidirectional Encoder Representations from Transformers (BERT),Generative Pre-Trained Transformers  (GPT) and Pathways Language Model (PaLM) after 2017.**

 5) Also, in the "Novelty" subsection, the last two sentences (starting with "Finally, we also ran...") are hard to understand. Could you please refine the writing --> **These two sentences were replaced by: Finally, we conducted a pilot phase for each step of the process. During this phase, a sample of papers (two for the data extraction step) was extracted and assessed by the reviewers. The results were then discussed, which allowed us to improve the proposed protocol and refine the data extraction template.**

 6) References contain many errors, such as wrong cases of titles --> **The references were all reviewed.**
